# The Contribution of the Maternal Immune System to the Establishment of Pregnancy in Cattle

**DOI:** 10.3389/fimmu.2015.00007

**Published:** 2015-01-28

**Authors:** Trudee Fair

**Affiliations:** ^1^School of Agriculture and Food Sciences, University College Dublin, Dublin, Ireland

**Keywords:** cow, fertility, macrophage, cytokine, immune function

## Abstract

Immune cells play an integral role in affecting successful reproductive function. Indeed, disturbed or aberrant immune function has been identified as primary mechanisms behind infertility. In contrast to the extensive body of literature that exists for human and mouse, studies detailing the immunological interaction between the embryo and the maternal endometrium are quite few in cattle. Nevertheless, by reviewing the existing studies and extrapolating from sheep, pig, mouse, and human data, we can draw a reasonably comprehensive picture. Key contributions of immune cell populations include granulocyte involvement in follicle differentiation and gamete transfer, monocyte invasion of the peri-ovulatory follicle and their subsequent role in corpus luteum formation and the pivotal roles of maternal macrophage and dendritic cells in key steps of the establishment of pregnancy, particularly, the maternal immune response to the embryo. These contributions are reviewed in detail below and key findings are discussed.

## Background

It is estimated that fetal viability is only achieved in about 55% of fertilizations in non-compromised cattle, indicating an embryonic/fetal mortality of about 35%. It is estimated that 70–80% of the total embryonic loss occurs between days 8 and 16 after insemination [day 16 corresponding to the day of maternal recognition of pregnancy; reviewed by Diskin and Morris (1)]. There are many reasons, related to both the mother and the embryo, why implantation fails but there is increasing interest in the role of the maternal immune system. Disturbed or aberrant immune function has been identified as primary mechanisms behind infertility. In contrast to the extensive body of literature that exists for human and mouse, studies detailing the immunological interaction between the embryo and the maternal endometrium in cattle have primarily focused on the role of the maternal recognition factor, the type I antiviral cytokine, interferon tau (2, 3) in corpus luteum (CL) maintenance, and progesterone priming of the endometrium. Nevertheless, by reviewing the existing studies and extrapolating from sheep, pig, mouse, and human data, we can draw a reasonably comprehensive picture of immune cell involvement from follicle development, ovulation, gamete transfer, maternal recognition of pregnancy, implantation, and placentation. These events are reviewed below and key findings are discussed.

## Ovarian Function

The presence and temporal regulation of neutrophils, eosinophils, macrophages (MΦ), granulocytes, and T-lymphocytes in ovarian tissues has been characterized extensively during the menstrual cycle in women; a smaller body of data exists for several farm animal species, including cows, sheep, pigs, buffaloes, and horses (4).

### Follicle differentiation

**Figure 1:**
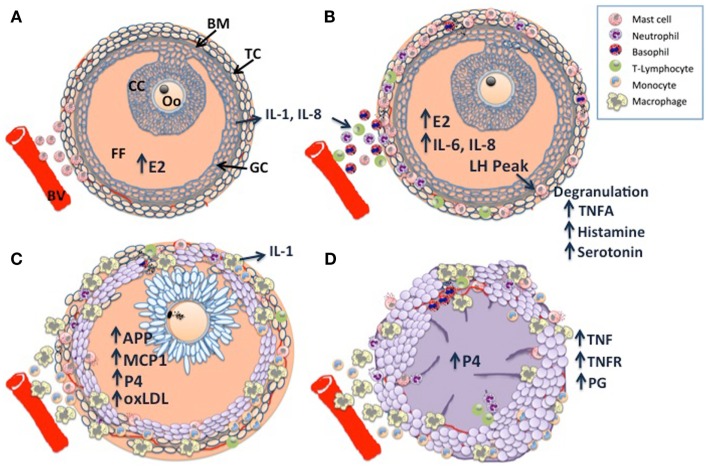
**Schematic diagram of dominant follicle differentiation and corpus luteum formation: follicle differentiation and luteinization appear to be characterized by three waves of immune cell infiltration**. **(A)** Mast cell infiltration of the thecal cell layer (TC) triggered by increasing estradiol (E2) concentrations, note the intact basement membrane (BM) separating the follicle granulosa-cell layer (GC) and follicle contents [cumulus cell layer (CC), surrounding the oocyte (Oo), and the follicular fluid FF] from the ovarian stroma. **(B)** A peak in luteinizing hormone (LH) pulsatility triggers mast cell degranulation which stimulates the second wave through the direct and indirect actions of TNF-alpha (TNFA), a constituent of the granules. **(C)** The last wave is characterized by macrophage infiltration, possibly in response to E2 and other chemoattractants such as monocyte chemoattractant protein 1 (MCP1), acute phase proteins (APP), and GC derived oxidized low density lipoprotein (oxLDL). Note the expanded cumulus cells surrounding the metaphase II stage oocyte, there is a switch from E2 synthesis to progesterone synthesis as the follicular cells become luteinized. **(D)** Following ovulation, granulocytes, neutrophils, and eosinophils constitute the majority of immune cells within the developing corpus luteum (CL), with further infiltration of macrophages and endothelial cells as development and vascularization proceed. Macrophage derived tumor necrosis factor (TNF) is a potent stimulator of luteal prostaglandins (PG), including PGF2a, PGE2, and PG112, which in concert with TNF drive CL vascularization. Large luteal cells derived primarily from the granulosa cells produce the >80% of P4.

Taken together, pre-ovulatory follicle differentiation and luteinization appear to be characterized by three phases of immune cell infiltration, which are illustrated in Figure 1: histological analysis of bovine dominant follicles shows that mast cell infiltration of the theca layer constitutes the first phase (5) (Figure 1A), luteinizing hormone (LH) triggered degranulation of the mast cells stimulates the second phase through the direct and indirect actions of TNF-alpha (TNFA), a constituent of the granules (Figure 1B). The second phase has been characterized in sheep and pigs as an influx of eosinophilic and neutrophilic granulocytes and T-lymphocytes (6, 7). The last phase of leukocyte migration consists of phagocytic monocytes (Mo); MΦ’s increase in the sow and ewe follicles at the time of ovulation (6, 7) (Figure 1C), possibly in response to peak estradiol concentrations (8). The temporal changes in the influx of leukocytes appear to occur in response to various chemoattractant cues produced by the developing follicle (9), indeed leukocyte chemoattractant activity has been demonstrated in bovine, ovine, and human follicular fluid of ovulatory follicles (10–12). Immunohistochemical characterization of the immune cell repertoire of the bovine ovary has largely focused on the formation and regression of the CL, which will be discussed later. In contrast, there are many reports detailing the transcriptomic profile of ovarian follicle development in cattle: (13–19). In particular, the deep sequencing analysis of bovine follicular theca and granulosa tissue during pre-ovulatory follicle development, revealed a profound effect of ovarian follicle stage on the expression of many genes within immune-related pathways in these tissues: during follicle differentiation, bovine thecal tissue was characterized by the expression of immune factors associated with vascularization, angiogenesis, and cellular proliferation (15, 20), processes which are carried out by MΦ’s in the theca layer during this time (21, 22). The bovine transcriptomic data also concurred with the histological findings described for sheep and pigs, as factors with known inflammatory/chemotactic properties such as *AKT2*, *ARHGEF1*, *GNAI2*, *IL-1*, *IL-6*, and *IL-8b* (23–25) were upregulated and pathways associated with MΦ and neutrophil function were overpopulated in differentiating thecal tissue (15).

### Follicle luteinization and ovulation

Findings from studies using rodent models indicate that the initiation of the ovulatory process occurs primarily in granulosa cells (26). Following the pre-ovulatory LH surge, morphological, endocrinological, and biochemical changes occur in the theca and granulosa cells, which redirect pre-ovulatory follicle development from differentiation to luteinization and thus the early stages of CL development (26). In particular, the post-LH deterioration of the basement membrane (BM) between the theca and granulosa-cell layers (GCs) (27), facilitates the movement of leukocytes into the granulosa tissue at luteinization, reflected by the peri-ovulatory granulosa-cell expression of factors involved in acute inflammation and immunosurveillance (15, 26, 28). It has been hypothesized that the dramatic increase in the expression of these signals in the follicle compartment activates the ovarian innate immune system (29) and that the damaged granulosa cells actively secrete alarmins or passively release them after death (30). Alarmins include acute phase proteins (APP), S100 proteins, advanced glycation end-products (AGE), high mobility group box-1 protein (HMGB1), defensins, and interleukin (IL)-1α, which are all present in follicle cells and the follicular fluid of pre-ovulatory follicles (26, 31–34) and can engage toll-like receptors (TLRs). In the case of ovarian granulosa cells, oxidized low density lipoprotein (oxLDL), which engages with TLR4 (34), has been proposed as a key alarmin in the pre-ovulatory cascade (29). This hypothesis is further supported by the identification of granulosa-cell exclusive expression of TLR signaling and NF-κB signaling pathways during luteinization in the bovine transcriptomic data (15). Furthermore, comparing the gene expression profiles of follicular tissue from heifers to that of lactating cows, it would appear that the recruitment of leukocytes to the differentiating follicle is delayed in cows. This is possibly a result of the demands of parturition/lactation in dairy cows, resulting in a reduced positive feedback loop, whereby lower steroid levels and chemoattractant signals recruit fewer leukocytes into the follicle, leading to lower steroid and chemoattractant levels (15).

### Corpus luteum formation

The CL is a transient organ established by cells of the follicle following ovulation; it is composed of a heterogeneous mixture of cell types that consist of not only steroidogenic luteal cells but also non-steroidogenic cells including vascular endothelial cells, fibroblasts, and immune cells such as lymphocytes and MΦ’s (35). Studies in human, rat and sheep indicate that the immune cells of the developing CL are recruited during ovulation (36–39) (Figure 1D), they were determined to have originated from the spleen (38), see Ref. (40), for review. Histological data from cattle indicate that they are primarily granulocytes, neutrophils, and eosinophils (35, 41, 42). However, as CL development and vascularization progresses, MΦ’s and endothelial cells infiltrate (43), providing a source of TNF and TNFR, the presence of which have been demonstrated in the bovine CL (44). TNF is a potent stimulator of luteal prostaglandins (PG) including PGF2a, PGE2, and PG12 (45), TNF and TNF-induced PGE2 have been proposed as key regulators of CL vascularization (46), recent work in the mare supports this hypothesis (47). Exposure to seminal plasma has been shown to enhance CL development and ovarian steroidogenesis: gilts treated with seminal plasma had heavier CLs, higher plasma progesterone (P4) levels, which peaked earlier, without a concurrent increase in ovulation rate, suggesting that the number and output of steroidogenic luteal cells is greater in animals exposed to seminal components (48). Immunohistochemical analysis revealed a greater abundance of predominantly major histocompatibility complex (MHC) class II positive MΦ’s and/or DCs in the stromal tissues and thecal cells of pre- and peri-ovulatory follicles, implying greater leukocyte recruitment at the time of ovulation in seminal plasma treated animals (49).

Immune function is central to CL regression, which must occur in the absence of pregnancy in order for new follicular development to take place (40). The regressing CL is characterized by an increase in MΦ and Mo populations, which eventually constitute the major proliferating cell type of the late regressing CL (40). The number of T-lymphocytes appears to increase just prior to the onset of luteolysis (35, 50), analysis of the bovine CL T-lymphocyte population revealed that 25% of T-lymphocytes present in a functional CL were T helper cells (CD4^+^), 45% were cytotoxic T-cells (CD8^+^), and 30% were gamma delta (γδ^+^) T-cells and that this profile did not alter during luteolysis (51). However, decreased P4 levels and interruption of growth factor signaling in the CL appear to promote both MΦ and T-cell activation, leading to increased TNF and INF production, respectively (52–55). TNF and INF are likely to be key regulators of apoptosis and ovarian tissue remodeling (56), their receptors are expressed in bovine steroidogenic cells and luteal cells (57). It is probable that Fas expression is induced in luteal cells by leukocyte-derived cytokines and that Fas L expressed on T-lymphocytes transduces apoptotic signals to the luteal cells [see Ref. (46), for review]. This is likely to be a conserved action as both Fas and Fas L are expressed in theca cells in multiple species (58).

## Gamete Transfer

### Inflammatory response to insemination

The site of semen deposition is very much a species-specific location (59). In cattle, and also in humans, sperm enters the cervix canal rapidly after semen deposition. The stimulation of vaginal insemination ensures the migration of neutrophils in to the cervical and uterine tissues (60, 61) and has been proposed as the initial point to optimize pregnancy success (62). The early immune response to insemination appears to contribute to both the ovulatory process and sperm cell selection; as reports from several species, including cattle, suggest that neutrophilic granulocytes target dead or capacitated sperm, thus removing non-motile or damaged spermatozoa (63–65), rather than motile, fertile sperm (62). In both humans and mice, it has been clearly demonstrated that the post-mating inflammatory response is mainly caused by the seminal plasma, with sperm having a negligible part (66). The cytokine, transforming growth factor-β (TGFβ), is the principal inflammatory trigger found in seminal plasma; it is primarily present within the male seminal plasma fluid in latent form, which is activated in the female reproductive tract by plasmin and other enzymes after insemination (62, 67). Although TGFβ itself can be chemotactic for a variety of immune cell types (68), in the murine uterus it was reported to act indirectly, by inducing cytokine and chemokine expression (69).

### Sperm transport

The delivery of seminal fluid to the female reproductive tract at coitus represents the first exposure of the female immune system to paternal alloantigens (62), raising the possibility that the female activates an immune response to male antigens in seminal fluid that may ultimately confer immunological tolerance to paternal antigens (70). This theory is supported by data from mice, which show that chemoattractants, secreted by eosinophils and neutrophils, attract both Mos and DC’s and shape the inflammatory status of MΦ’s (71, 72). The response is not restricted to vaginal exposure; intrauterine horn insemination was shown to induce recruitment of MHC class II positive cells in gilts (73). Seminal plasma contains estrogen and testosterone, PG, and various signaling molecules, including IL-8, TGFβ, and IFNG, as well as bacterial lipopolysaccharide (LPS) (62). When murine uterine and cervical cells come into contact with the constituents of semen, they are stimulated to synthesize and release granulocyte-macrophage colony-stimulating factor (GM-CSF), IL-6, and further chemokines (66, 74), which stimulate MΦ’s, DC, and granulocyte infiltration of the uterine and cervical tissues (75). The induction of IL-6 is required for TGFβ to induce the generation of IL-17 producing, pro-inflammatory TH-17 cells, which in turn favor the induction of neutrophil-chemotactic IL-8 (76). In conjunction with IL-8, TGFβ induces the secretion of pro-inflammatory cytokines such as IL-1B, IL-6, and leukemia inhibitory factor (LIF) (77). Although, the expression of TGFβ was shown to increase in the bovine endometrium during the implantation period (78), the relatively high pregnancy rates achieved in cattle following artificial insemination or embryo transfer undermines the importance of maternal exposure to seminal plasma in cattle. The findings of studies designed to address this point indicate that neither exposure to seminal plasma nor TGFβ is critical for to the establishment of pregnancy in cattle (79, 80).

### Immune tolerance post-fertilization

Exposure to paternal antigens occurs in two waves in the reproductive process: initially during transmission of seminal fluid at coitus, and secondly when placental trophoblast cells come in contact with maternal tissues during embryo implantation (81). In sheep and cattle, morula-stage embryos enter the uterus around day 4–5 and blastocysts are formed by day 6 and 7, respectively, hatching occurs within 48–72 h. The hatched blastocyst subsequently elongates reaching 10 cm or more in length by day 14 and 25 cm or more in length by day 17 and the conceptus trophectoderm and endometrial luminal epithelium (LE) become closely apposed, see Ref. (82), for review. Implantation is a superficial, protracted affair in these species, commencing after attachment and adhesion of the trophectoderm to caruncular and intercaruncular areas on day 16 in sheep and day 19 in cattle. Again, in contrast to the volume of data that has been acquired in human and mouse studies, the number of investigations carried out in farm animal species on the involvement of the maternal immune system in the establishment of pregnancy is very limited, particularly, for early pregnancy. For several decades, human pregnancy was described as a Th1/Th2 dichotomy with an imbalance toward a Th2 type immune response (83, 84). However, this paradigm is considered a simplistic explanation of the molecular events occurring during pregnancy, as it does not account for reported endometrial expression of Th1-type cytokines during implantation (85, 86). In ruminants, studies investigating maternal immunomodulation by pregnancy have focused on the actions of the type 1 interferon, IFNT, which is secreted by the elongating conceptus and is the main signaling factor in maternal detection/recognition of pregnancy (87, 88). Initial studies demonstrated that endometrial luminal epithelial cell estrogen receptor and oxytocin receptor expression was down regulated in response to IFNT (89, 90). Critically for the continuation of pregnancy in cattle, this binding eventually results in the attenuation of endometrial PGF2a secretion, allowing CL production of P4 to be maintained (90). In addition to its anti-luteolytic properties, IFNT appears to be the key regulator of the maternal immune response in ruminants (91, 92), acting on the endometrium to induce or enhance the expression of genes hypothesized to regulate uterine receptivity to implantation and conceptus development (78, 93–95). The expression of IFNT is limited to the embryonic trophectoderm during the peri-implantation period (96). Additionally, there is significant evidence that the bovine conceptus does not endeavor to conceal itself immunologically, as MHC-I transcripts have been detected in early cleavage stage bovine embryos (97) and in first and second trimester and term trophoblast tissues (98). Furthermore, *MHC* class I mRNA expression by bovine embryos is both transcript- and embryo stage-specific (97) and can be regulated by a number of cytokines including IFNG, IL-4, and LIF (99, 100).

## Maternal Recognition and Response to Pregnancy

### Monocytes, macrophages, and dendritic cells

Macrophage recruitment to the pregnant endometrium occurs in a wide range of mammalian species, including the mouse (101), human (102, 103), cynomolgus and vervet monkeys (104), sheep (105), and cattle (78, 106, 107). While their role has not been completely elucidated, functions include clearing of apoptotic cells, regulation of apoptosis (108), and regulation of placental lactogen concentrations at the fetal–maternal interface (109). Given the potential antigenicity of the conceptus due to paternal antigen and classical MHC protein expression (97), MΦ’s may also feature in curtailing the activation of anti-conceptus immune responses (106). In cattle, the maternal immune response to the developing embryo is characterized by the expansion of Mo, MΦ’s (CD14^+^-cells), and DC (CD172a–CD11c^+^) populations in the endometrial stroma as early as day 13 of pregnancy (78). Interestingly, there was a parallel decrease in CD11b^+^-cells; CD11b is associated with Mo movement through the endothelium, which would imply that the Mo had acquired a stationary phenotype (78).

Dendritic cells have been shown to play an important role in decidua formation and the induction of immune tolerance in human and murine pregnancy (110, 111). Employing individual and combined CD172a and CD11c labeling of the bovine endometrium, it was determined that there was a high prevalence of immature cells within the endometrial DC population during early pregnancy (78). Immature DC’s have been associated with the initiation and maintenance of peripheral tolerance (112) and their presence in large numbers in the uterine decidua has been associated with the establishment of healthy pregnancies in women (113). It is most likely that in cattle, IFNT induces this initial maternal response to the presence of the elongating embryo, either by attracting monocytes into endometrium or by modulating their differentiation into MΦ’s or DC. Indeed, gene expression analysis of the same endometrial tissue revealed dramatic up-regulation of mRNA expression of IFN stimulated genes *IL12B*, *MCP1*, *MCP2*, *PTX3*, *RSAD2*, *ISG15*, and *TNFA* (78). Furthermore, MCP1 and MCP2 are members of the cellular chemoattractant chemokine β subfamily, which have highly potent MΦ recruitment and activation properties (114), thus increased *MCP1* and *MCP2* expression may be associated with the recruitment of Mo/MΦ from the systemic system into the endometrium. The up-regulation of the evolutionary conserved PTX3 is very interesting; gene deletion studies in mice have shown that it is essential for female fertility, participating in the assembly of the cumulus oophorus extra-cellular matrix (115). Moreover, PTX3 is involved in innate immunity, proposed roles include selected pathogen recognition, opsonization leading to enhanced phagocytosis, regulation of the inflammatory response, complement-mediated clearance of apoptotic cells, and control of autoimmunity (116–118).

### T-lymphocytes

Despite the evidence from studies in humans and mice linking successful pregnancy with an imbalance toward a Th2 immune response type, data from cattle indicate that CD4^+^, CD8^+^, γδTCR^+^, and FoxP3 T-lymphocyte populations are not regulated temporally during estrus or early pregnancy in cattle (119). However, mRNA expression analysis on the same tissue revealed that the Th1 immune factors *IFNA*, *LIF*, *IL1B*, *IL8*, and *IL12A* were down regulated during the luteal phase of the estrus cycle, whereas the Th2 factors LIF and IL10 were upregulated, suggesting that the phenotypes/inflammatory status of Th cells are tightly modulated during the estrous cycle in anticipation of pregnancy. Additionally, LIF and IL-10 have been shown to regulate MΦ activation (120, 121). Similarly, endometrial *TGF*β*2* expression is down regulated during the ovine and bovine implantation period and is subsequently increased during placentation (78, 122), which may reflect TGFβ2 involvement in Mo recruitment and regulation of MΦ inflammatory status (123). Furthermore, the study in cattle showed TGFβ localization to the fetal–maternal interface of the bovine placentome, which may indicate TGFβ2 involvement in restricting trophoblast invasion during the implantation phase, while enhanced expression during placentation and *in vitro* cell culture studies, suggest that TGFβ2 may play a mitogenic role during placentation, promoting caruncular growth, and coordinating epithelial cell development leading to placentome formation (123, 124).

Surprisingly, and in contrast to the situation in human and mouse models, where NK cells can constitute up to 70% of the endometrial lymphocyte population during the preimplantation phase of pregnancy (112), when uterine NK (uNK) cells are believed to play a pivotal role in local vascular remodeling and regulation of trophoblast invasion [for review see Ref.(125, 126)]; NK cells do not appear to play such a critical role during early pregnancy in cattle. Indeed, the only published data suggests the bovine endometrium population of CD335^+^ NK cell population is not expanded as an immediate response to maternal recognition of pregnancy (119). The findings of an *in vitro* study which demonstrated anti-proliferative effects of recombinant IFNT exposure on immune and uterine cells, particularly leukocytes, infers that the IFNT secretion by the embryo may actively restrict NK cell expansion in early pregnancy (127), which is in keeping with the non-invasive nature of implantation in cattle [see review by Bazer et al. (128)]. However, further studies are required to determine if the NK cell population expands when IFNT secretion wanes and to what degree, if any, they are involved in placentation.

## Concluding Remarks

Intensive cattle production systems have been associated with postpartum immunosuppression and subsequent reduced fertility; it is vital that basic research in the area of bovine reproductive immunology is expanded to generate new knowledge by which these issues can be overcome. However, although the number of studies investigating the contribution of the maternal immune system to reproductive function in cattle is a fraction of that carried out in human and mouse species, it is possible to conclude that maternal macrophage and dendritic cells play pivotal roles in key steps of the establishment of pregnancy, particularly, development of the CL and maternal immune response to the embryo.

## Conflict of Interest Statement

The author declares that the research was conducted in the absence of any commercial or financial relationships that could be construed as a potential conflict of interest.
